# Seizure forecasting: Where do we stand?

**DOI:** 10.1111/epi.17546

**Published:** 2023-03-08

**Authors:** Ralph G. Andrzejak, Hitten P. Zaveri, Andreas Schulze‐Bonhage, Marc G. Leguia, William C. Stacey, Mark P. Richardson, Levin Kuhlmann, Klaus Lehnertz

**Affiliations:** ^1^ Department of Information and Communication Technologies Universitat Pompeu Fabra Barcelona Spain; ^2^ Department of Neurology Yale University New Haven Connecticut USA; ^3^ Epilepsy Center, Neurocenter University Medical Center, University of Freiburg Freiburg Germany; ^4^ Department of Neurology, Department of Biomedical Engineering BioInterfaces Institute, University of Michigan Ann Arbor Michigan USA; ^5^ Division of Neurology VA Ann Arbor Medical Center Ann Arbor Michigan USA; ^6^ School of Neuroscience Institute of Psychiatry Psychology and Neuroscience, King's College London London UK; ^7^ Department of Data Science and AI, Faculty of Information Technology Monash University Clayton Victoria Australia; ^8^ Department of Epileptology University of Bonn Medical Centre Bonn Germany; ^9^ Helmholtz Institute for Radiation and Nuclear Physics University of Bonn Bonn Germany; ^10^ Interdisciplinary Center for Complex Systems University of Bonn Bonn Germany

**Keywords:** monitoring devices, multimodal monitoring, network theory of epilepsy, quality of life, seizure control, seizure cycles, seizure prediction, seizure risk, wearables

## Abstract

A lot of mileage has been made recently on the long and winding road toward seizure forecasting. Here we briefly review some selected milestones passed along the way, which were discussed at the International Conference for Technology and Analysis of Seizures—ICTALS 2022—convened at the University of Bern, Switzerland. Major impetus was gained recently from wearable and implantable devices that record not only electroencephalography, but also data on motor behavior, acoustic signals, and various signals of the autonomic nervous system. This multimodal monitoring can be performed for ultralong timescales covering months or years. Accordingly, features and metrics extracted from these data now assess seizure dynamics with a greater degree of completeness. Most prominently, this has allowed the confirmation of the long‐suspected cyclical nature of interictal epileptiform activity, seizure risk, and seizures. The timescales cover daily, multi‐day, and yearly cycles. Progress has also been fueled by approaches originating from the interdisciplinary field of network science. Considering epilepsy as a large‐scale network disorder yielded novel perspectives on the pre‐ictal dynamics of the evolving epileptic brain. In addition to discrete predictions that a seizure will take place in a specified prediction horizon, the community broadened the scope to probabilistic forecasts of a seizure risk evolving continuously in time. This shift of gears triggered the incorporation of additional metrics to quantify the performance of forecasting algorithms, which should be compared to the chance performance of constrained stochastic null models. An imminent task of utmost importance is to find optimal ways to communicate the output of seizure‐forecasting algorithms to patients, caretakers, and clinicians, so that they can have socioeconomic impact and improve patients' well‐being.

**Key points**

- Multimodal monitoring is possible over periods of many months, and there is first evidence for the additional predictive value of information from effectors of the autonomic nervous system provided by peripheral sensors.
- There is no universal feature extracted from signals measured from the patient for seizure forecasting; rather the best features differ for different patients.
- Network‐based approaches to the human epileptic brain provide novel and promising insights into forecasting and controlling seizures.
- Multidien cycles lasting from days to months can be used to design algorithms that forecast the probability of seizure occurrence at a 24‐h horizon.
- Both discrete seizure warnings and probabilistic seizure‐risk forecasts offer different benefits to patients, caretakers, and clinicians.
- It is essential to clearly define the stochastic model and all underlying assumptions when testing whether a seizure‐forecasting algorithm works better than chance.
- Clinicians should be educated and trained in how to interpret seizure forecasts and in advising patients and caretakers how to respond to the forecast.

## 1 INTRODUCTION

Whereas seizures appear to occur unpredictably for patients, they do not occur randomly, and the search for precursors at the level of behavioral manifestations and brain dynamics has become a major field of research. Patients may have pre‐ictal prodromes subjectively experienced or observed,^1^ and several groups have reported a better‐than‐chance patient‐based prediction of upcoming seizures,^2^ suggesting the existence of a pre‐ictal state that may be used to predict an upcoming seizure. This motivates a search for more objective and independent measures of brain function to forecast seizures. The conceptual understanding of ictogenesis driving research on seizure forecasting is that in addition to the inter‐ictal, ictal, and post‐ictal states, there exists a distinct pre‐ictal state. Although progress has been made in the past three decades,^3^, ^4^ until recently primarily through time‐series analysis of electrophysiological measurements, a definition of the pre‐ictal state and the nature of the transition between background activity and seizure continues to elude investigators. There are a number of reasons for this, ranging from the initial conceptual proposal for a pre‐ictal state, one which was not grounded on an understanding of physiological mechanisms; the lack of accepted conceptual, computational, or animal models for ictogenesis; the logistical challenges of studying an event with a low probability of incidence within a controlled setting; and the difficulty of capturing the serial dynamics entailed in seizure onset with modalities other than electrophysiology.

Research that addresses these multiple challenges has allowed a glimpse into the nature of the pre‐ictal state, illuminating it with observations that both refine our understanding of ictogenesis and raise additional questions.^5^, ^6^, ^7^ A decade ago, the first prospective human trial demonstrated prospective seizure forecasting.^8^ Multiple studies have reported changes that discriminate between the inter‐ictal and pre‐ictal state at various scales of neuronal organization from the local to the network level. This further provides evidence that epilepsy is a large‐scale brain network disease that requires novel approaches to improve understanding of the network's aberrant structure and function.^9^ New approaches with minimally invasive and non‐invasive devices providing multimodal predictive biomarkers and conducted over prolonged periods are of interest for broader application. These new devices are expected to aid the capture of both direct and surrogate measures of the pre‐ictal state and to better define the transition from the background state to the ictal state and to open new areas for investigation. The observation of multiday (multidien) rhythms in epilepsy poses new challenges for the design and execution of neuroscience and clinical neuroscience studies to understand this phenomenon and its relationship to seizure generation, and to incorporate this new information within a definition of the pre‐ictal state.^10^

Formulating seizure forecasts relies on the notion that patients can benefit from discrete warnings ahead of an impending seizure, but also from probabilistic seizure‐risk estimates that can guide subjective decision‐making about daily planning. Quantifying the performance of these forecasts is critical to ensure that they are clinically and commercially useful. Regardless of whether the forecast is issued as a discrete warning or as a seizure‐risk estimate and regardless of the applied metrics, it remains essential to test whether the employed algorithm performs better than what can be expected by chance. There cannot be a unique definition of chance level. Instead, the field faces the challenge to formulate a battery of well‐defined null hypotheses, underlying stochastic models, and assumptions to quantify the performance expected for a variety of chance levels. These strategies must then be organized and presented in a fashion that allows patients, caretakers, and clinicians to engage, understand, and tailor the results to their own preferences.

## 2 MULTIMODAL SEIZURE FORECASTING

Multimodal assessment in patients with epilepsy has become available in an outpatient setting using wearable and implantable devices. With such devices, almost continuous ultra‐long‐term electroencephalography (EEG) data (over many months to years) and long‐term data on motor behavior (using electromyography [EMG], accelerometry, and global positioning system [GPS] data) and signals of the autonomic nervous system (heart rate, skin conductance, and skin temperature) can be obtained unobtrusively and without patient stigmatization. These multimodal data sets can be used for objective seizure documentation,^11^ to identify circadian and multidien seizure cycles,^12^ and as the basis for forecasting periods of increased seizure propensity.^13^

Multimodality was first implemented in the groundbreaking study of,^8^ using long‐term intracranial EEG recordings and acoustic recordings to objectively verify seizures. This study showed the need for objective seizure measures, as patient‐based seizure documentation had no significant correlation with actual seizure occurrence (See also Ref. ^14^). Autonomic features like pre‐ictal electrocardiography (ECG) have been shown to provide information that is almost as predictive as intracranial EEG recordings, suggesting a relevant role of non‐EEG data obtained with peripheral sensors.^15^, ^16^, ^17^

Using the multimodal biosensor wristband E4 (Empatica), assessing raw data from accelerometry, skin conductance, blood volume pulses, and skin temperature in 69 patients undergoing in‐patient video‐EEG monitoring, a statistically significant performance of seizure forecasting was found in 43% of patients using deep‐learning techniques.^18^ With a mean prediction horizon of 30 min, time in warning in the significant performers was 43.7%, and sensitivity for correct seizure prediction was 75.6%. Notably, longer duration of recordings improved forecasting performance, pointing toward possible improvements when using ultra‐long recordings. It is notable that leaving out any of the four modalities decreased the performance of seizure forecasting, pointing to non‐redundant information on pre‐ictal information obtained from different sensors.

First ambulatory studies, with small numbers of subjects, suggest significant prediction performance with either multimodal E4 data in patients with simultaneous intracranial data from responsive neurostimulation devices^19^ or from subcutaneous ultra‐long EEG recordings.^20^, ^21^ The combination of such signals obtained with low invasivity over periods of months may open up new windows to assess the spectrum of pre‐ictal physiological alterations,^12^ and to make use of machine‐learning methods using extensive training data to obtain clinically useful forecasting performances.^22^

## 3 GENERAL FEATURE EXTRACTION IN SEIZURE FORECASTING

As noted earlier, multimodal biosignals are used for seizure forecasting, but how do seizure‐forecasting algorithms use these data? The predominant approach is to extract signal features or characteristics from the multimodal time‐series, typically using a sliding analysis window such that feature time‐series can be extracted.^4^ These feature time‐series are then investigated for their ability to differentiate pre‐ictal from inter‐ictal, ictal, and post‐ictal states.^23^ As a result, seizure‐forecasting algorithms can be devised by analyzing the feature time‐series most capable of differentiating the pre‐ictal state from other states.^3^, ^24^

The multiple signal features investigated have been either univariate, bivariate, or multivariate, that is, involving single‐, two‐, or multiple‐channel analysis, respectively. Signal features have been derived from time‐domain, frequency‐domain, linear and nonlinear signal analysis, or graph theoretical analysis.^3^, ^4^, ^23^, ^25^, ^26^, ^27^ Although a very large number of features have been tested for seizure forecasting, there is no single feature that is a reliable biomarker for the pre‐ictal period; rather the best forecasting performance is usually obtained when a specific combination of features is selected in a patient‐specific fashion. Often machine‐learning algorithms are used to perform this feature combination process, and in some cases, machine learning alone is used to extract and combine features from raw data.^3^, ^25^, ^26^

Much of the early analysis of signal features for seizure forecasting was performed with short‐term EEG recordings lasting at most 2 weeks per patient and containing only a handful of seizures.^4^ The short duration of these data sets meant that long time‐scale analysis windows were not available, so it was challenging to obtain a robust estimate of the performance of forecasting algorithms. This challenge was addressed by introducing long‐term implanted devices, such as one that allowed the first‐in‐man trial of a seizure‐forecasting algorithm,^8^ which demonstrated the feasibility of feature‐based seizure forecasting. Furthermore, the commercialization of long‐term closed‐loop seizure‐control devices^28^ has made it possible to obtain long‐term data sets of short epochs of intracranial, mostly pathophysiological, EEG and corresponding feature detections for a decade or longer in individual patients. Although this only partially meets the community's guidelines,^3^, ^4^ it has nevertheless opened the door for seizure forecasting based on long time‐scale analysis windows that are providing a deeper understanding of seizure cycles.^10^, ^24^, ^29^, ^30^

## 4 CYCLES IN SEIZURE FORECASTING

Seizure occurrences in epilepsy have always been hypothesized to have a cyclic nature. The earliest studies describing circadian^31^ and longer timescale^32^ organization of seizures appeared early in the last century. Over the past decade, the introduction of chronic EEG devices allowed monitoring and studying of long cycles in focal epilepsy in an unprecedented way. Using the Neurovista^8^ and the Neuropace^33^ devices, researchers found multidien (multiday, from days to months) variations of the inter‐ictal epileptiform activity.^10^, ^29^ These findings have been confirmed in cohorts of 222 patients,^34^ with different biosignals,^35^ and even in other species such as dogs.^36^ Moreover, the strength of these multidien cycles and their relationship to seizures appear to be comparable to circadian cycles.^34^

All this new evidence has opened the possibility of using the multidien cycles to forecast seizures in a horizon that was thought to be unachievable until a few years ago. Indeed, as it was shown in a retrospective study, it is possible to use the phases of the multidien cycles to output probabilistic forecasts in a 24‐h horizon,^30^ where 66% of subjects have an improved forecast over chance as evidenced by seizure time surrogates.^37^ Similar results can also be obtained using minimally invasive devices.^18^, ^38^, ^39^ In addition, these multidien are similar across subjects, allowing the development of generalizable models that forecast seizures to new subjects.^40^, ^41^ In conclusion, the discovery of these long cycles and their applications to forecast seizures over long horizons (24‐h up to days in advance) has shifted the overall mindset of the community from searching the pre‐ictal state (minutes before the seizure) to forecasting the pro‐ictal state (days with high seizure risk).^35^, ^42^

## 5 BRAIN NETWORKS IN SEIZURE FORECASTING

Considering the brain as a complex network, with its structure and dynamics evolving in time,^43^, ^44^ has led to a paradigm shift in establishing epilepsy as a large‐scale network disorder.^9^, ^45^, ^46^, ^47^ Graph‐theoretical concepts and methods^27^ then enable improved characterizations of such networks. Early network‐based seizure‐prediction studies^48^, ^49^, ^50^, ^51^ have reported limited predictive performance from temporal changes of global network characteristics that assess the network's functional segregation, integration, or robustness. This can be ascribed to various biological rhythms that distinctly affect brain networks.^52^ In contrast, a well‐above‐chance‐level^37^ predictive performance was reported recently for temporal changes of characteristics of single network constituents,^53^, ^54^, ^55^ based on which network mechanism for the epileptic brain's transition into the pre‐ictal state was proposed. The transition is characterized by a rearrangement of the larger epileptic network's path structure, which results in a formation of bottlenecks that impairs physiologic brain communication—an important factor in brain disorders.^56^ Such a backbone‐like substructure emerges from brain regions (network nodes) that are usually deemed unaffected by the focal epileptic process but that act as bridges (groups of network links) between remote regions, that is, far off the seizure‐onset zone. An in‐depth investigation of the bridging dynamics is currently underway and employs novel, refined analysis techniques.^57^ This can lead to an improved understanding of the complex spatial–temporal interaction phenomena preceding seizures in the large‐scale epileptic brain network that cannot be captured by, for example, early warning indicators designed for simple one‐dimensional systems.^58^, ^59^, ^60^ Using another network‐based approach to characterize the aforementioned pre‐ictal phenomena, a measure of brain resilience was developed recently.^61^ Counterintuitively, resilience was increased in the hours before most of the investigated seizures. This may hint at a reduced effectiveness of antiseizure medication in drug‐resistant focal epilepsies or reflects the epileptic brain's ability to efficiently defy control by virtue of its intrinsic plasticity and adaptiveness.^62^ In summary, network‐based seizure forecasting provides novel insights into the pre‐ictal dynamics of the evolving epileptic brain as well as important clues for optimizing existing interventions and the development of novel therapeutic interventions for controlling seizures.

## 6 HOW TO FORMULATE A FORECAST AND HOW TO QUANTIFY ITS PERFORMANCE?

As touched on above, various characteristics can be derived from multimodal data (intracranial/scalp EEG, wearables, and so on) to formulate seizure prediction or forecasting algorithms.^3^, ^4^ These algorithms can be based on raw signal characteristics or leverage machine learning to analyze multiple characteristics at once to formulate seizure predictions or forecasts.^26^ The traditional approach has been to threshold the time series of a characteristic to generate predictions. More modern approaches feed multiple characteristics derived from the raw data into machine‐learning algorithms, the output of which can be thresholded to generate predictions. Such all‐or‐nothing predictions/warnings are not the only option. The probability of a seizure occurring within a certain amount of time can also be obtained conditionally on the data‐derived characteristics or via the outputs of machine‐learning algorithms.^63^ Such probabilities enable the possibility of seizure forecasts.^30^, ^63^ These probabilities themselves can also be thresholded to generate multiple seizure‐risk levels (e.g., low, moderate, and high)^8^ that could be used to simplify the interpretation of raw probabilities, which on their own would need to be interpreted subjectively by the patient or clinician.

Once seizure predictions or forecasts have been generated, there are various ways that their accuracy can be evaluated against the true seizure time series. Seizure predictions can be evaluated through the calculation of different metrics like sensitivity, specificity, area under the receiver‐operating characteristic curve and time in false warning.^3^, ^4^ Seizure forecasts can be evaluated by other metrics such as Brier scores and calibration curves.^63^ It is important to note that these metrics, in concert with robust statistical analysis, can be used to objectively compare different seizure prediction or forecasting algorithms in the search for clinically useful algorithms.

## 7 FORECASTING BETTER THAN CHANCE—WHAT CHANCE?

An early lesson in seizure forecasting was to recognize the need to test whether the applied technique performs above chance level.^37^, ^64^ Two decades later, variations of the chance level definition continue to defy comparisons between studies.^24^ On the other hand, using different complementary chance level definitions can actually be key to increasing the confidence in a study's observation. Assume, for example, that a binary predictor (yes/no) issues warnings at random times with some minimal interval between subsequent warnings. Now we test the null hypothesis that the predictions are issued by a Poisson process. If enough data are available, we will correctly reject this wrong null hypothesis. The Poisson process has no memory and, therefore, there is no minimal interval between subsequent warnings. However, this does not mean that the predictor works better than chance. It simply means that the stochastic model underlying our null hypothesis is not the one underlying the random warnings.

It is, therefore, essential to precisely formulate the null hypothesis about the stochastic model producing the output of the forecasting algorithm. All assumptions underlying the model should be stated, since the violation of any of them will lead to the rejection of the null hypothesis. Neglection thereof might render an above‐chance performance inconclusive. Of equal importance is exploiting the flexibility of the null hypotheses. Only after testing and rejecting several null hypotheses, each comprising different assumptions, does one have converging evidence that the forecasting algorithm works better than chance.

In some cases, the forecasting performance expected under the null hypothesis can be calculated by analytical means.^64^, ^65^, ^66^, ^67^ Greater flexibility can be achieved by applying constrained randomizations to the original data to numerically estimate this expected value.^37^, ^68^, ^69^ As performed, for example by Proix et al.,^30^ such numerical approaches should be applied even if the performance metrics have well‐defined analytical chance levels, like 0.5 as area under the receiver‐operating characteristic curve. This can help to rule out that deviations from the metric's chance level are due to some hidden bias in the forecasting and evaluation algorithm.

## 8 HOW TO COMMUNICATE THE FORECAST TO THE CLINICIAN?

Smartphone applications such as the Seer App now provide seizure‐risk estimates for users based upon a patient's own seizure diary.^70^ At the current time, there is no evidence to inform how such risk estimates might be shared with the clinician caring for the person with epilepsy, or how the clinician should respond. It is likely that the clinician will need to play a key role in guiding and educating the patient about how to respond. Therefore, it is important that the clinician should be well informed about how to interpret the forecast. In addition, we can expect that clinicians may not wish to receive a continuous seizure‐risk estimate or every seizure warning generated by a prediction system.

Interpretation of a probabilistic forecast is likely to be challenging. For example, in a scenario requiring obstetricians to interpret a probabilistic forecast of the risk of Down syndrome following a screening test, they were mostly incorrect in their interpretations.^71^ Moreover, when probabilistic information is provided from multiple cues, the cognitive strategy used by different individuals to make a judgment about whether a future event will occur is variable^72^ and altered by common disorders such as anxiety.^73^ Ensuring an appropriate clinician response to a probabilistic forecast is also likely to be challenging. In other areas of medical practice, early warning systems have been developed that provide a probabilistic prediction that a patient is at risk of imminent deterioration. Despite clear protocols, evidence shows that appropriate treatment escalation may not be undertaken due to a range of educational, organizational, and cultural factors.^74^, ^75^

An efficient way to incorporate clinician advice alongside a seizure forecast might be to anticipate the broad categories of advice that a patient might seek and incorporate this advice automatically alongside the forecast. For example, in a system to predict future changes in blood glucose level in people with diabetes, simple advice is included about how to respond to the prediction; retrospective evaluation showed that inclusion of advice alongside the forecast led to improved glycemic outcomes.^76^

## 9 HOW TO COMMUNICATE THE FORECAST TO THE PATIENT AND CARETAKER?

The methods we described represent decades of academic progress, but the translation to patients has been extremely limited. The first trial included 15 patients in Australia and was funded by a startup company specifically formed to create the first seizure prediction device.^8^ The data acquired from that trial have been transformative in epilepsy research; yet despite its success (and the disappointment of several of those patients), that company dissolved after the trial and the device was explanted. This first attempt at seizure forecasting has served as both a model and a warning to the field. One roadblock was that regulatory agencies were not familiar with how to assess a “warning device.” How can one justify the risk of a clinical trial if there is “no treatment”? What constitutes success or failure? Although the field of seizure forecasting continued to progress in the research lab, these questions blocked further trials and prevented any further progress in patients for several years.

Interest in revisiting a clinical trial was rekindled when the Epilepsy Foundation of America sought to answer these questions from an overlooked source: the patients themselves. Community engagement is now increasingly recognized as a critical part in the development of new technologies such as this, in order to determine the acceptable tradeoff between risk and benefit.^77^ The “My Seizure Gauge” Initiative^78^ surveyed patients with epilepsy and their caregivers, who stated that the uncertainty of when a seizure will occur is one of the most concerning issues they face.^79^ Later surveys revealed that patients preferred wearable devices over implanted ones, identifying times with high risk over low risk and shorter warning times (3–5 min) to reduce stressful waiting periods, and that patients were more tolerant of inaccurate predictions than their caregivers.^80^ Patients overwhelmingly think such a device is “extremely important,” would use it, and would change their immediate plans based upon the forecast. On the other hand, most patients are quite concerned about potential monetary cost.^81^

These results must now also be interpreted in the modern context of wearable technology. Many patients are already receiving various forms of health statistics about sleep quality, heart rate variability, and even possible seizures from non‐invasive devices such as standard smartwatches. Such information has not undergone clinical trials and it is not regulated; yet it has become the norm. Patients are adjusting their behavior based upon suggestions from their watch or phone: sleep longer, take a day off exercise, or get up to walk for a few minutes. With these messages, consumers have become more aware of how technology can help them and more tolerant of the variability—and perhaps fallibility—of the data and predictions. This environment presents an excellent opportunity for seizure forecasting. Wearables typically do not provide dogmatic diagnoses but rather gentle coaxing and suggestions, which fits much better the idea of forecasting an increased risk, rather than “predicting” that a seizure must occur in the imminent future. Consumers also quickly learn that their wearables are not 100% accurate but they use them anyway. Within the field of seizure forecasting, researchers, regulators, and investors have previously been reluctant to accept such inaccuracies. But it is now clear that patients want *something* and are learning how to use information even if it is not perfect, much like a weather forecast. If we can provide an efficient warning system, presented in a way that can coax patients to safe behaviors or reduce stress during safe periods, the patients will use it. Then instead of struggling over how to quantify its success, we can simply ask the patients whether the information has improved their quality of life.

## 10 CONCLUDING REMARKS

Although several milestones have been passed toward the accurate seizure forecasting, many more need to be achieved in the years to come. This brief review started with a reminder of seizure anticipation by patients and it closes by clarifying that the ultimate measure of the benefit of seizure forecasting is the patient's quality of life. The next stages on the long and winding road toward seizure forecasting at clinically useful levels would benefit from further improving our knowledge of seizure generation as well as from a greater understanding of common goals and better communication between the scientific community, patients, caretakers, and clinicians.

## AUTHOR CONTRIBUTIONS

All authors conceptualized the review, drafted the manuscript, and edited the manuscript into its final version.

## CONFLICT OF INTEREST STATEMENT

W.S. has a licensing agreement with Natus Medical, Inc, and a consulting agreement with Neuronostics, Inc. Neither Natus nor Neuronostics were involved in the content of this paper. Neither of the remaining authors has any conflict of interest to disclose. We confirm that we have read the Journal's position on issues involved in ethical publication and affirm that this report is consistent with those guidelines.
